# A Study on the Frequency-Domain Black-Box Modeling Method for the Nonlinear Behavioral Level Conduction Immunity of Integrated Circuits Based on X-Parameter Theory

**DOI:** 10.3390/mi15050658

**Published:** 2024-05-17

**Authors:** Xi Chen, Shuguo Xie, Mengyuan Wei, Yan Yang

**Affiliations:** School of Electronic and Information Engineering, Beihang University, Beijing 100191, China

**Keywords:** integrated circuit (IC), models of integrated circuits for RF immunity behavioral simulation-conducted immunity modeling (ICIM-CI), immunity modeling, electromagnetic compatibility (EMC) modeling, direct power injection (DPI), X-parameters

## Abstract

During circuit conduction immunity simulation assessments, the existing black-box modeling methods for chips generally involve the use of time-domain-based modeling methods or ICIM-CI binary decision models, which can provide approximate immunity assessments but require a high number of tests to be performed when carrying out broadband immunity assessments, as well as having a long modeling time and demonstrating poor reproducibility and insufficient accuracy in capturing the complex electromagnetic response in the frequency domain. To address these issues, in this paper, we propose a novel frequency-domain broadband model (Sensi-Freq-Model) of IC conduction susceptibility that accurately quantifies the conduction immunity of components in the frequency domain and builds a model of the IC based on the quantized data. The method provides high fitting accuracy in the frequency domain, which significantly improves the accuracy of circuit broadband design. The generated model retains as much information within the frequency-domain broadband as possible and reduces the need to rebuild the model under changing electromagnetic environments, thereby enhancing the portability and repeatability of the model. The ability to reduce the modeling time of the chip greatly improves modeling efficiency and circuit design. The results of this study show that the “Sensi-Freq-Model” reduces the broadband modeling time by about 90% compared to the traditional ICIM-CI method and improves the normalized mean square error (NMSE) by 18.5 dB.

## 1. Introduction

**Figure 1:**
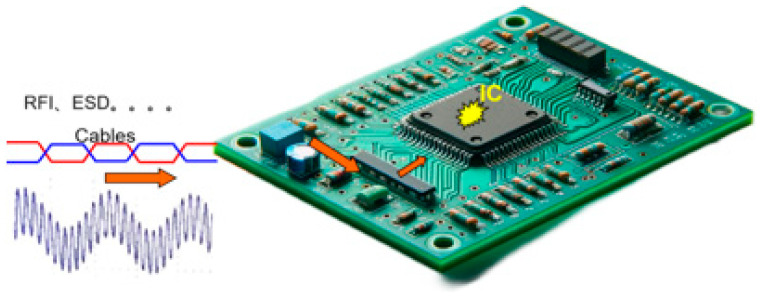
External interference causes sensitization of the chip on the board.

The immunity of integrated circuits is related to the electromagnetic safety of electronic devices (Figure 1) [1]. Researchers J. Loeckx and G. Gielen [2] found that small circuit topology changes can increase the immunity of integrated circuits by several orders of magnitude during DPI testing [3]. In light of this finding, being able to predict whether a device will pass sensitivity testing before it is manufactured is of great importance in terms of cost reduction [4,5]. However, chip manufacturers in general do not provide immunity models for their chips; as such, circuit designers must obtain a model of the chip’s behavior by utilizing specific testing methods. This behavioral model is called the “black-box model”, and involves extraction without knowing the internal physical details of the chips; instead, through the mapping of input and output signals, an abstract mathematical expression is obtained to describe the relationship between the recorded input signal x(t) and the output signal y(t). When the black-box model and the DUT are inspired by the same input signal, the output of the black-box model should effectively be as close as possible to the actual response of the DUT. The use of behavioral models protects the intellectual property (IP) rights of the device manufacturer and reduces the difficulty involved in modeling [6]. The authors of articles [7] and [8] used artificial neural networks (ANNs), Volterra levels [9], time-domain behavioral models such as envelope domain models [10], nonlinear impulse response models [11], and two-path memory models [12] in their studies, and constructed time-domain-sensitive behavioral models of devices to describe the output behavioral effects of the devices. However, time-domain models usually require a large number of tests to be conducted and for characterizations in the time domain to be made in order to generate a model that is accurate over a specific frequency range [13]. In the aforementioned techniques, the models can only be used to focus on IC faults (detection efficiency, jitter, etc.) in the time domain, and it is difficult to analyze mismatches between ports and high harmonics, which in turn prevents them from meeting the needs of broadband EMC applications. Moreover, one model only supports the simulation of a single frequency point under a single interference, and a large number of simulation models need to be established if the simulation is performed in the broadband frequency range. Therefore, frequency-domain models are more suitable for simulating distributed components over a bandwidth. In recent years, the ICIM-CI [14] model has been shown to predict chip immunity. The ICIM-CI [15] model is able to approximate and replace accurate chip or circuit simulations by using a look-up table and power distribution network (PDN)-based approach and enables the use of the model in the frequency domain [16].

However, the model still needs to depend on time-domain measurements when obtaining the IB (input behavior) netlist, which leads to the need for a large number of tests in broadband multi-frequency applications as well as redundant designs, which makes the process cumbersome and time-consuming. This then leads to a lack of model adaptability and the need to rebuild the model frequently when the criterion is changed, which in turn increases the amount of work required and overall time consumption. In addition, the ICIM-CI model has limitations in handling the nonlinear characteristics of the chip when it is under interference, and linear assumptions often need to be made [17], which leads to discrepancies between the model and the actual measurement results. In addition, since the model is based on the go–no-go decision criterion, it is not capable of parametric simulation, which limits its ability to accurately predict and meticulously analyze the performance of integrated circuits in complex electromagnetic environments and also obstructs circuit designers from integrating the chip into the cascade simulation of the whole circuit board. Due to this issue, the model is unable to comprehensively assess the electromagnetic characteristics of the circuit, resulting in a negative impact on overall design efficiency. Therefore, although the ICIM-CI model provides a framework for frequency-domain analysis, its limitations in terms of efficiency and nonlinear characteristic handling [18], as well as its inability to support overall quantitative simulation at the board level, are important challenges that need to be overcome.

In this study, we present the “Sensi-Freq-Model”, a novel immunity black-box model for the rapid frequency-domain characterization of chips based on X-parameter theory, which aims to address the limitations of existing models. The model is accurate in characterizing the output characteristics of chips in both time and frequency domains and supports the parametric simulation of chips in circuits. The main advantage of the model is that it can adapt to changes in the sensitivity criteria so that circuit designers can quickly adapt to changes in design specifications or complex electromagnetic environments without having to dedicate long periods of time to reconstructing the model, which greatly improves the model’s versatility and modeling efficiency. This flexibility and efficiency are especially important for modern circuit design, in particular in the pursuit of high-precision and fast iterative engineering.

The present paper is organized as follows: Section 2 describes the structure and theoretical basis of the model. Section 3 illustrates the extraction process and simulation results through two simulation examples. Lastly, in Section 4, we verify the modeling results and analyze them through the use of a test modeling example.

## 2. Sensi-Freq-Model Structure

**Figure 2:**
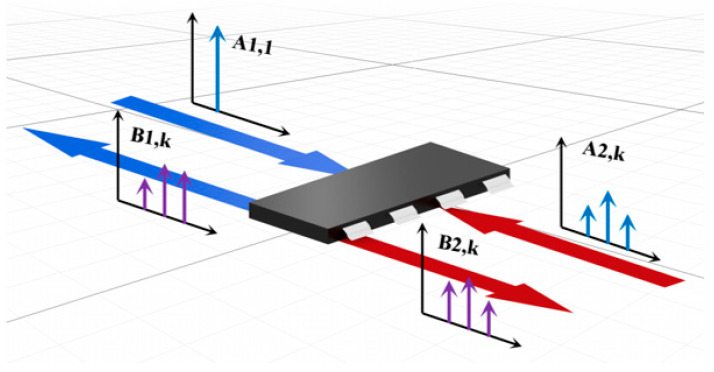
Schematic diagram of multi-harmonic spectrum mapping for a two-port network. Blue color represents inputs and outputs of port 1; red color represents inputs and outputs of port 2.

In general, chips exhibit nonlinearity when sensitized by conducted interference [19], and this nonlinear effect is the main cause of EMC failures on chips. This is why the assumption of linearity often leads to biased final predictions [20,21]. When a small signal is injected, at this moment, the system exhibits linear characteristics and the harmonic frequencies at the output are negligible. The behavior of the chip can be sufficiently characterized using the scattering parameters in this case; however, with an increasing number of injected signals, the system will exhibit nonlinearity, and the range available for the scattering parameter will continue to decrease. The harmonic response cannot be ignored (Figure 2), at which point the output signal will produce multi-harmonic spectral mapping on the chip. Therefore, based on the characteristics of the chip-conducted interference response, the Sensi-Freq-Model modeling theory is proposed in combination with the X-parameter theory [22,23].

We introduce a set of multivariate complex functions $F_{pk}(.)$ into the frequency domain that relate all relevant input spectral components $A_{qn}$ to the output spectral components $B_{pk}$ (where q and p range from 1 to the number of signal ports and m and n range from zero to the highest harmonic index). The mathematical expression is as follows:

(1) $$B_{pk}=F_{pk}\left(A_{11},A_{12}\ldots ,A_{1n},A_{21},A_{22},\ldots ,A_{2n},...A_{q1},A_{q2},...A_{qn}\right)$$

where $A_{11}$ denotes the input interference fundamental frequency.

While the chip is under the interference condition, it usually comprises a large-signal interference input component $A_{11}$, a DC excitation component, a regular drive signal input component, and other input components (harmonic frequency components). At this time, the relatively small regular drive signal input component satisfies the superposition principle, and all of the large-signal excitation and large-signal response in the excitation can be expressed by Equation (2):

(2) $$LSOP=\{\begin{array}{l} DCS^{\left(LOSP\right)}=\left\{DCS_{q}\right\}\\ RFS^{\left(LOSP\right)}=A_{11}\\ DCR_{p}^{\left(LOSP\right)}=X_{p}^{\left(FDCR\right)}\left(\left\{DCS_{q}\right\},\left|A_{11}\right|\right)\\ B_{p,k}^{\left(LOSP\right)}=X_{p,k}^{\left(F\right)}\left(\left\{DCS_{q}\right\},\left|A_{11}\right|\right)P^{k} \end{array}$$

where ${DCS}^{\left(LSOP\right)}$ denotes the DC excitation present in the chip related to the large-signal DC bias excitation ${DCS}_{q}$ at port *q*.

${RFS}^{\left(LSOP\right)}$ denotes the RF interference excitation present in the chip equal to the large-signal interference input component $A_{11}$.

${{DCR}_{p}}^{\left(LSOP\right)}$ denotes the DC response at the large-signal operating point of port *p* related to the large-signal DC bias excitation ${DCS}_{q}$ at port *q*, and the large-signal interference input component $A_{11}$, with the parameter $X_{p}^{\left(FDCR\right)}$ used to denote the X-parameter element of the excitation portion of the DC bias voltage.

$B_{p,k}^{\left(LSOP\right)}$ denotes the system response at the large-signal operating point related to the large-signal DC bias excitation ${DCS}_{q}$ at port *q* and the large-signal interference input component $A_{11}$, with the parameter $X_{p,k}^{\left(F\right)}$ used to denote the X-parameter element of the large-signal operating point’s influence [24].

Therefore, combining the effects of the large-signal nonlinear mapping and the linear non-analytic mapping that describe the co- and cross-frequency disturbances caused by the small-signal incidence, the scattering wave at the response port can then be described by Equation (3):

(3) $$\begin{array}{ll} B_{pk} & ≅X_{pk}^{F}(refLOSP_{in})P^{k}+\sum_{\begin{array}{c} q=1\\ l=1\\ \left(q,l)\neq (1,1\right) \end{array}}^{\begin{array}{c} q=N\\ l=K \end{array}}X_{pk,ql}^{S}(refLOSP_{in})P^{k-l}A_{ql}\\ & +\sum_{\begin{array}{c} q=1\\ l=1\\ \left(q,l)\neq (1,1\right) \end{array}}^{\begin{array}{c} q=N\\ l=K \end{array}}X_{pk,ql}^{T}(refLOSP_{in})P^{k+l}A_{ql}^{*} \end{array}$$

where $P=e^{j\varphi (A_{11})}$ is a unit-length phase quantity with the same phase as $A_{11}.X_{pk}^{F},X_{pk,ql}^{S}$, and $X_{pk,ql}^{S}$ represent frequency-domain X-parameter elements that describe co-frequency disturbances caused by a small signal incident on the port of the device during testing. $X_{pk,ql}^{T}$ represents a frequency-domain X-parameter element that describes the cross-frequency disturbances caused by a small signal incident on the port of the device during testing. LSOPin represents the excitation portion of the LSOP, and refLSOPS represents the corresponding reference excitation.

The A-wave of incidence and the B-wave of scattering have two sets of indexes: p and q refer to the port number of the chip, while k and l refer to the number of harmonics.

## 3. Results

### 3.1. Immunity Modeling Based on Simulated Circuits

**Figure 3:**
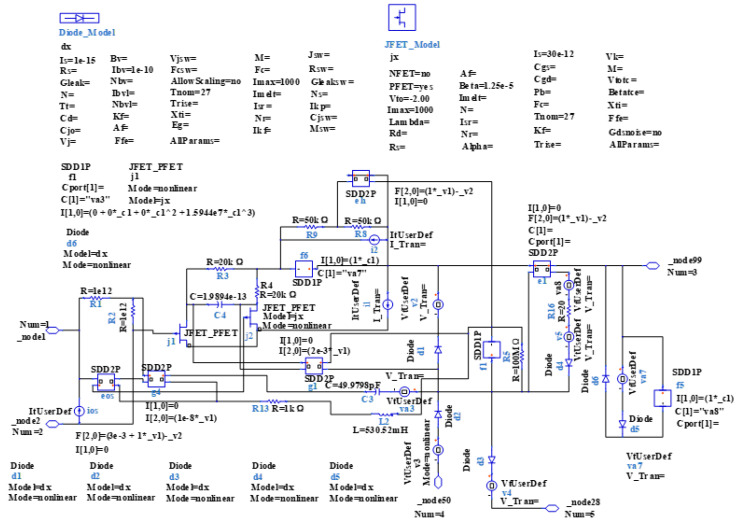
The constructed op-amp white-box model is used to extract the Sensi-Freq-Model and verify its accuracy. Component labeling in blue, component parameters in black.

**Figure 4:**
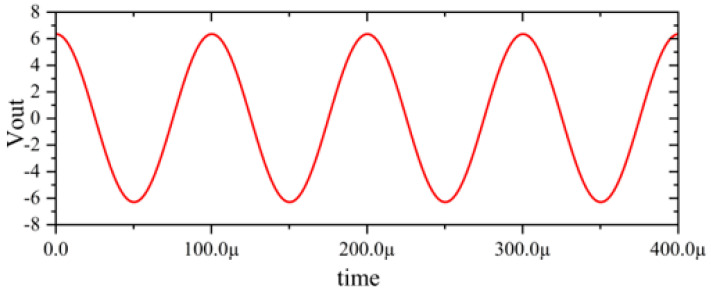
Performance without interference.

**Figure 5:**
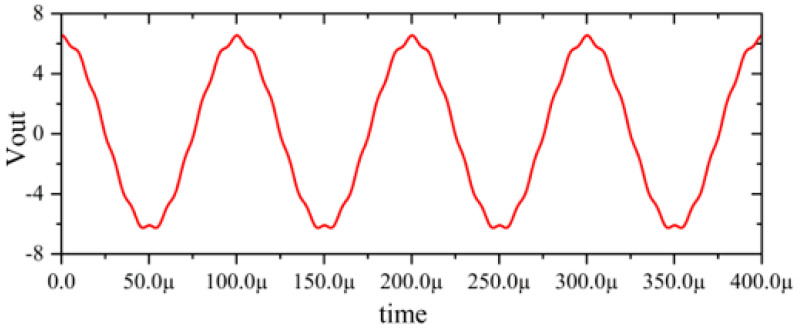
Slight jitter in the output waveform when subjected to −25 dBm interference, not exceeding the tolerance requirements.

**Figure 6:**
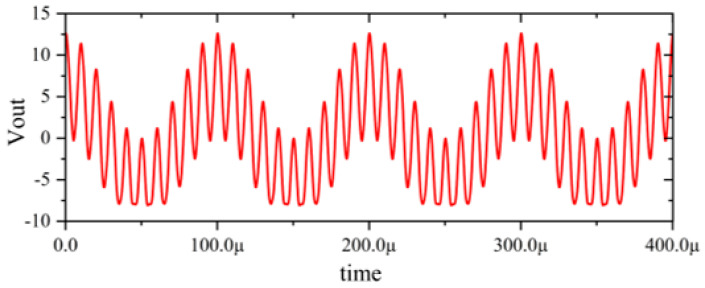
Jittery output waveforms exceeding tolerance requirements when subjected to 0 dBm interference.

**Figure 7:**
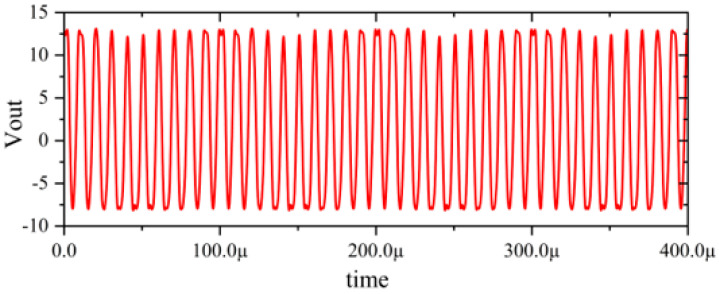
Severe jitter in the output waveform exceeding the tolerance requirement when subjected to 10 dBm interference.

In this section, a method for extracting immunity models using chip-simulated circuits is presented and the accuracy of the Sensi-Freq-Model is verified. An analog circuit model of an operational amplifier built based on white-box theory is used. The circuit structure and specific parameters are shown in Figure 3. This white-box model was built to extract the Sensi-Freq-Model and verify the difference between its output in the simulation software and the white-box model. The op-amp is a forward amplifier circuit, and the parameters set for normal operation are ${Vin}^{+}$: f=10 kHz, V=100 mV sine wave signal, and the DC bias voltage set to $V^{+}=+15\;V$ and $V^{-}=-10\;V$. When the chip is in normal operation, the output of the circuit is as seen in Figure 4. Based on the DPI test method of IEC62132-4 [3], interference signals of different frequencies and powers are applied to the op-amp’s input ports and power supply ports. The interference signals are selected for continuous waveforms according to Section 5 of IEC 62132-4. Here, the interference noise signal is simulated using the signal source module (power source-N Frequencies and Power Levels) in the ADS (advanced design system). The output of the op-amp is shown in Figure 4, Figure 5, Figure 6 and Figure 7. Interference signals of different levels, when observing the output response sensitivity characteristics of the monitoring port, can be mainly divided into four phenomena (Table 1), which can be used to describe the faults as four types of situations according to the IC performance level specified in IEC62132-1 [25]. The specific description is shown in Table 1, and its output waveform schematic is shown in Figure 4, Figure 5, Figure 6 and Figure 7.

Through the use of simulation, the Sensi-Freq-Model is extracted, a simulation model is built, the accuracy of the model is tested and it is verified as to whether the model can accurately reflect the output response of the chip under different disturbed situations to compare the built Sensi-Freq-Model with the traditional ICIM-CI model in the frequency-domain immunity prediction curves.

#### 3.1.1. Model Extraction

**Figure 8:**
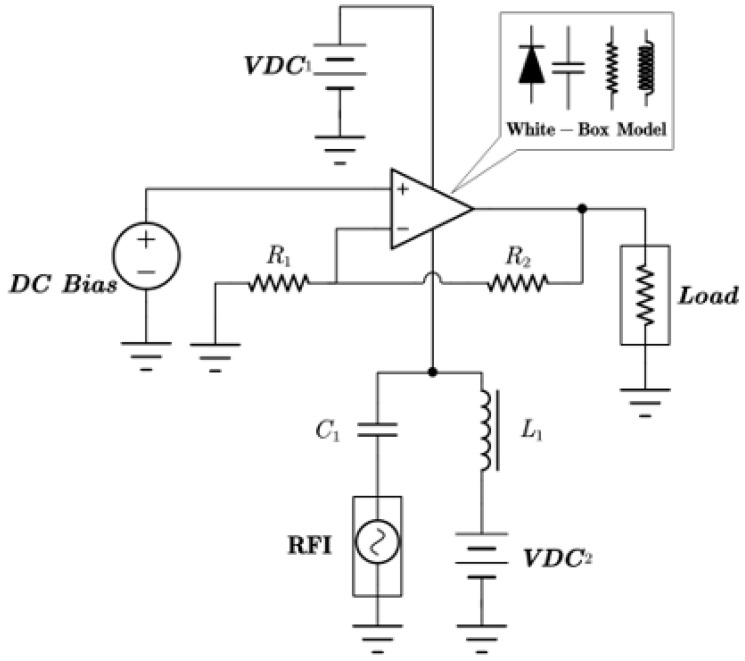
Extraction of injection immunity models for the power terminal of V−. *DC* Bias is a *DC* source; *R*_1_ and *R*_2_ are resistors; *C*_1_ is the capacitor; *L*_1_ is the inductor; Load is the matching load; RFI is a source of radio frequency interference; *VDC* is the *DC* voltage source for the amplifier.

**Figure 9:**
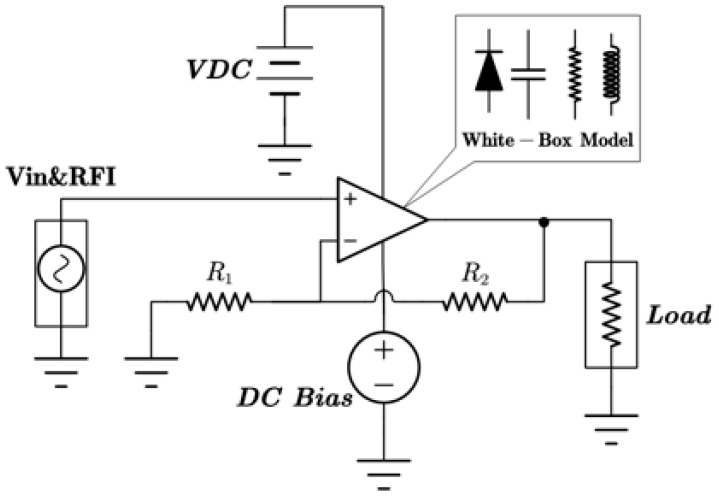
Extraction of the injected immunity model for the input terminal of Vin+. *DC* Bias is a *DC* source; *R*_1_ and *R*_2_ are resistors; Load is the matching load; Vin and RFI are the amplifier’s function signal and interference signal injection source; *VDC* is the *DC* voltage source for the amplifier.

During normal operation of the chip, the interference injection signal is injected from V− and Vin+ (Figure 8 and Figure 9) separately, and the power of the interference signal is in the range from −40 dBm to 20 dBm. Each parameter in Equation (2) is solved according to the proposed method outlined in Section 2 to complete the operational amplifier immunity model.

#### 3.1.2. Model Verification

**Figure 10:**
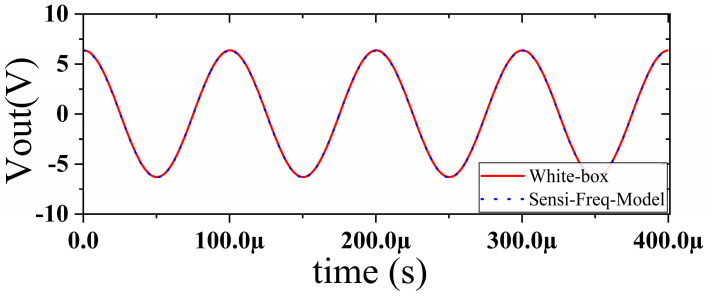
Comparison of simulation model output and actual output results during normal operation of the chip.

**Figure 11:**
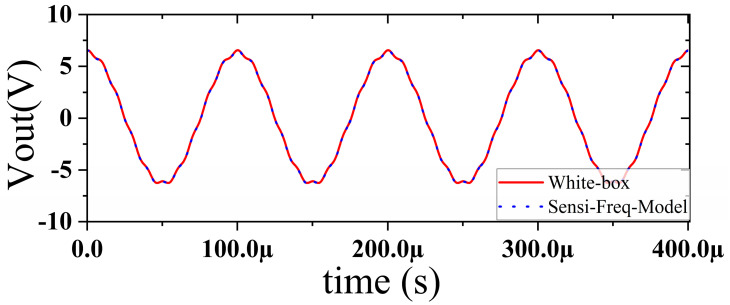
Comparison of simulation model output and actual output when slight jitter occurs at the chip output (interference injection −25 dBm).

**Figure 12:**
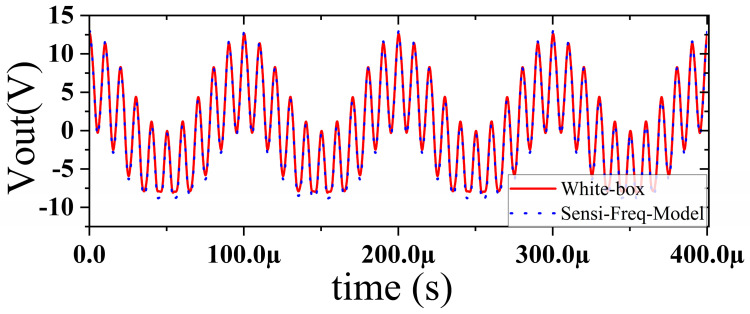
Comparison of simulation model output and actual output results when severe jitter occurs at the chip output (when interference is injected at 0 dBm).

**Figure 13:**
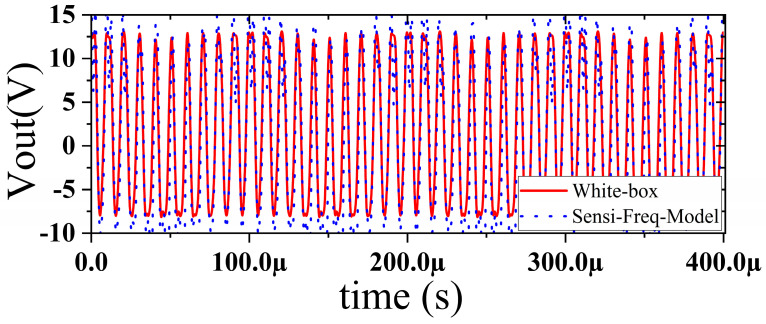
Comparison of simulation model output and actual output results when the chip output is severely disturbed (interference injection at 10 dBm).

The accuracy of the extracted immunity model is examined by first verifying whether the model can output an accurate time-domain response waveform at a single frequency. As an example, the frequency of interference injection to the input is set at 100 kHz to verify the accuracy of the model. It can be seen that the model is able to accurately simulate the response waveforms of the device under various disturbed/unperturbed states, such as normal operation (Figure 10), distorted output waveform (Figure 11), distorted and jittered output waveform (Figure 12), and severely distorted output signal (Figure 13). These results show that the model is able to accurately predict the disturbed behavior and provide quantitative waveforms.

#### 3.1.3. Discussion

**Figure 14:**
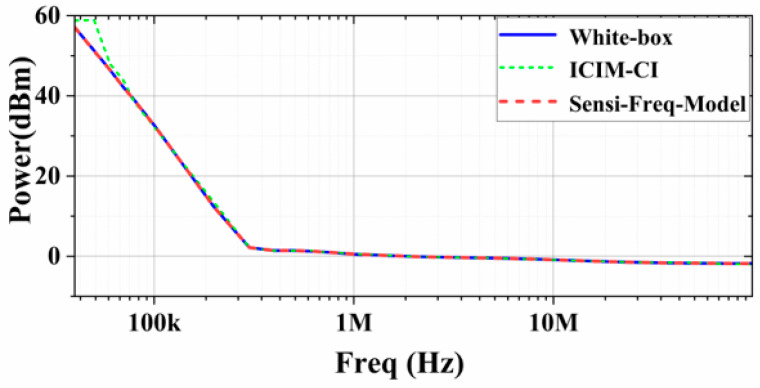
Comparison of the sensitivity prediction of the two modeling methods with the actual white-box output results (at the power supply side’s V− with the immunity criterion at ΔVout_p-p_ ≤ 5%).

As can be seen from the results displayed in the above section, the Sensi-Freq-Model can directly output the response waveform of the chip after being perturbed; therefore, it is only necessary to build broadband immunity prediction curves for different immunization standards after one test. Compared to the traditional ICIM-CI model, which needs to determine the immunity criteria before establishing the immunity prediction curves, the Sensi-Freq-Model does not need to be re-modeled due to the change in the test criteria, which will result in significant savings in overall modeling time. In this section, the immunity criterion is set to the condition that the allowable change in peak-to-peak output voltage ΔVout_p-p_ is ≤5% for comparison purposes, and Figure 14 shows the comparison of the results of using the method proposed in this paper and the traditional ICIM-CI modeling method with white-box simulation when interference is injected from the power supply side through the V- port. It can be seen that, under this immunity criterion, both modeling methods predict the sensitivity better because the chip has higher linearity.

**Figure 15:**
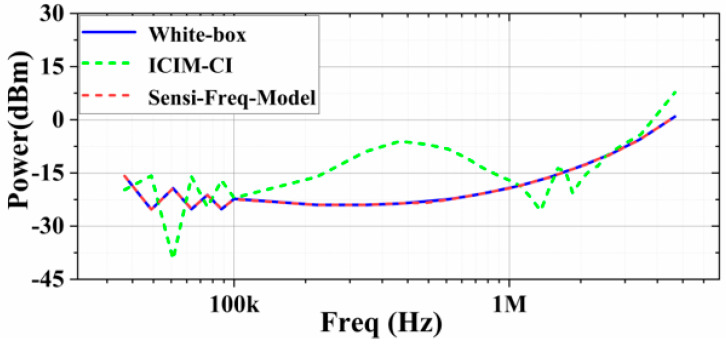
Comparison of the sensitivity prediction of the two modeling methods with the actual output results (at the Vin+ port with the immunity criterion at ΔVout_p-p_ ≤ 5%).

When injecting interference to Vin+ and setting ΔVout_p-p_ ≤ 5%, it can be seen that the prediction accuracy of the ICIM-CI model deteriorates when the system has a nonlinear response due to interference; in contrast, the Sensi-Freq-Model’s prediction accuracy is still relatively satisfactory (Figure 15).

**Figure 16:**
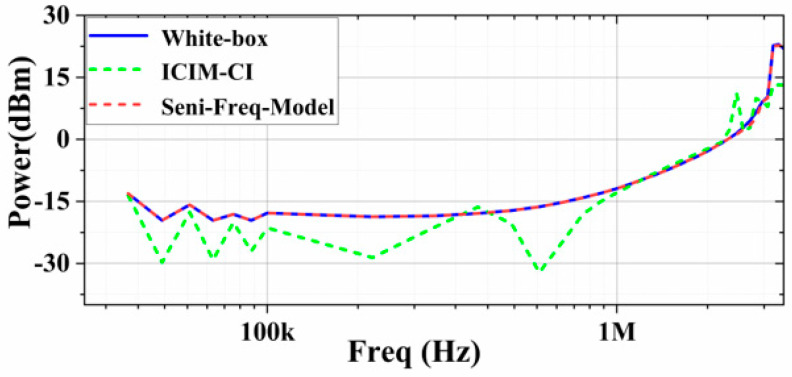
Comparison of the sensitivity prediction of the two modeling methods with the actual white-box output results (at the Vin+ port with the immunity criterion at ΔVout_p-p_ ≤ 10%).

Changing the immunization criterion to output voltage peak-to-peak ΔVout_p-p_ ≤ 10%, the ICIM-CI requires that the model be rebuilt; however, the Sensi-Freq-Model can provide the immunity curve directly based on the output waveforms, and it can also be seen that the prediction accuracy of ICIM-CI is still lower than the prediction accuracy of the Sensi-Freq-Model (Figure 16).

It can be seen that the Sensi-Freq-Model can provide a very accurate output response in both time and frequency domains. Compared to ICIM-CI, even when the immunity criterion is changed, the model can still accurately predict the sensitivity phenomena of the device under examination without the need for re-measurement and modeling, which will greatly reduce the time required for modeling and testing.

### 3.2. Immunity Modeling Based on Measurements

In this section, we will verify the accuracy of the methodology by obtaining a Sensi-Freq-Model immunity model of the device using actual instrumentation and performing immunity simulations using the model. An operational amplifier, which is more susceptible to sensitization, was chosen for testing and modeling. The amplifier was used in a voltage follower configuration [26], in which interference to the input differential pair may cause the amplifier output to be offset, making the amplifier inoperable [27]. In addition, of all the possible interference signals, those overlaid on the op-amp input pins are the most difficult to prevent [28]. The op-amp is powered by a ±2.5 V supply voltage, with the V+ pin set to 0 VDC. Interference signals are injected through a bias tee on this pin.

Using a test instrument to extract the Sensi-Freq-Model parameters of the chip when it encounters a sensitive injection, the achieved immunity model of the chip is then loaded in the simulation software, and the generated model simulation results are compared with the output generated using the DPI test.

#### 3.2.1. Model Extraction

**Figure 17:**
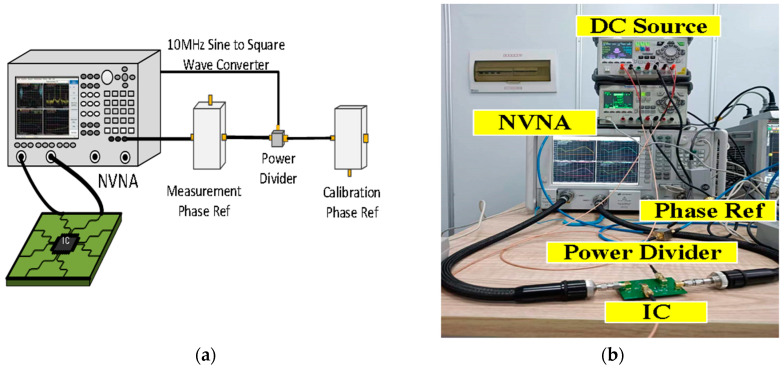
Model extraction test setup. (**a**) Test setup schematic; (**b**) Actual test set-up.

The Sensi-Freq-Model of the chip can be extracted using a nonlinear vector network analyzer (NVNA), signal source (optional), DC source, external phase reference generator, and appropriate instrument control and processing software [29]. The NVNA provides the RF interference environment to which the chip is exposed. The DC source provides the chip’s basic operating environment, and an external phase reference generator is used to provide a standard phase reference to ensure phase consistency. The setup configuration is shown in Figure 17.

Using the test setup described above, the parameters of Equation (3) can be solved and expressed as a matrix, enabling the chip’s Sensi-Freq-Model to finally be generated. The model parameters of this operational amplifier are extracted from the 10–100 MHz band under RF interference from −10 dBm to 10 dBm. We loaded the Sensi-Freq-Model in the simulation software and performed a two-port harmonic balance simulation to simulate the behavior of the op-amp when exposed to interference.

#### 3.2.2. Model Verification

**Figure 18:**
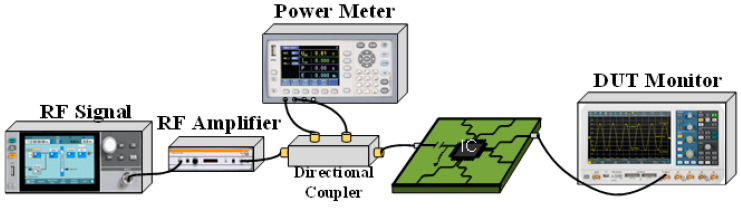
DPI test setup.

The results obtained from the DPI measurements were compared with the equivalent model obtained to verify the accuracy of the model, and the DPI test configuration is shown in Figure 18.

By using an RF generator, RF amplifiers, directional coupler, bias-tees, RF power meters, oscilloscopes, and other equipment, the interference waveforms specified in IEC62132-1 are applied to the chip, and the output waveforms of the chip are recorded. DPI measurements are performed by injecting an interference signal into the DUT (on the V+ pin or input) via a bias tee, with the operational amplifier used as a follower circuit and powered by a supply voltage of ±3 V. The V+ pin is set to 0 VDC.

**Figure 19:**
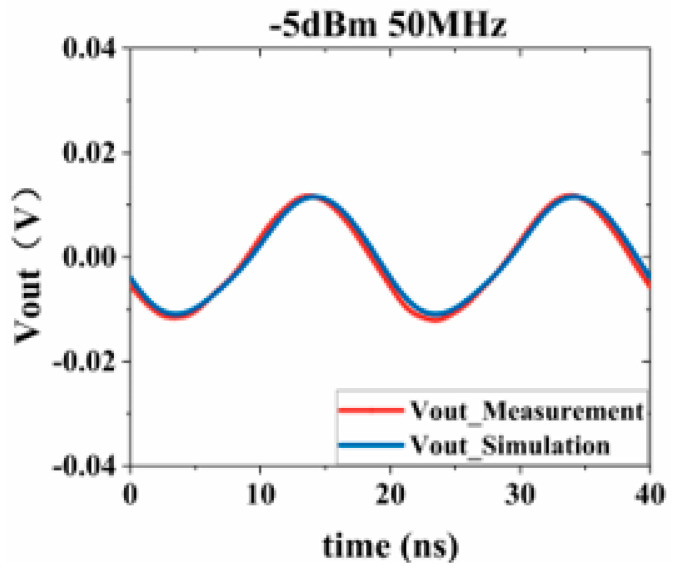
Injection of −5 dBm interference.

**Figure 20:**
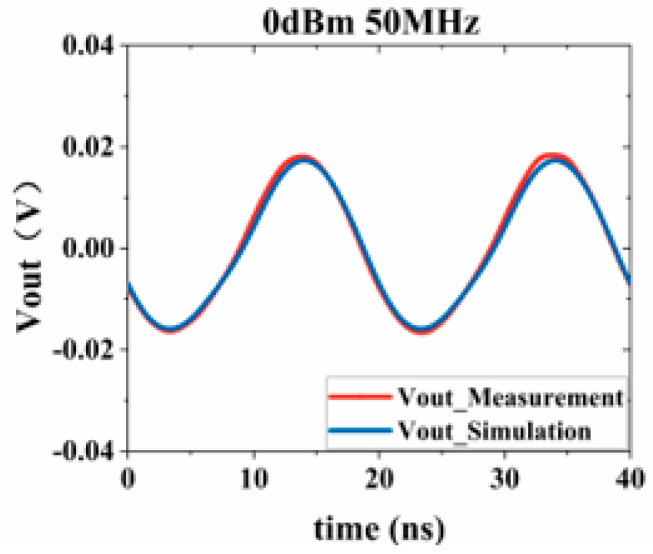
Injection of 0 dBm interference.

**Figure 21:**
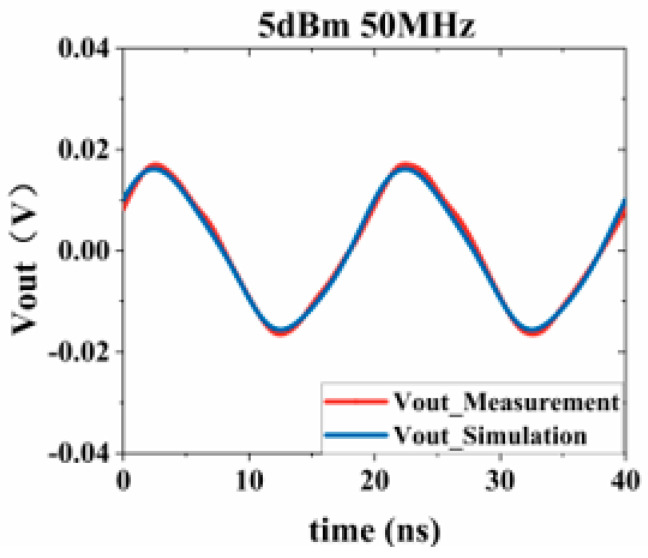
Injection of 5 dBm interference.

**Figure 22:**
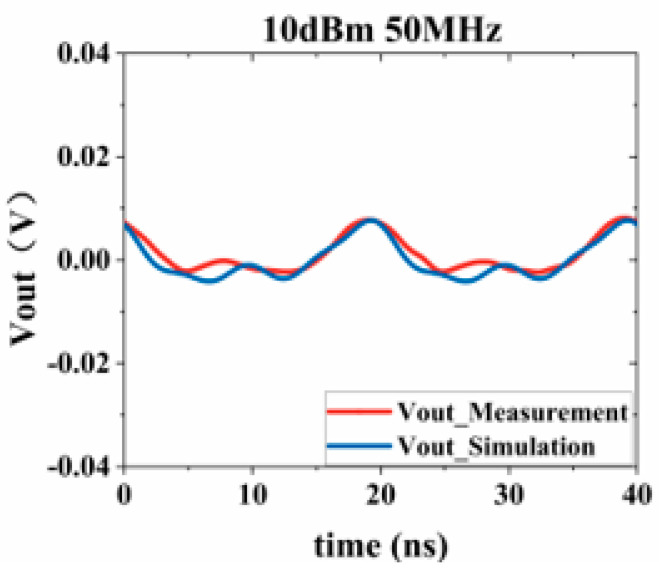
Injection of 10 dBm interference.

Substituting the test results into Equation (3), the response values of the Sensi-Freq-model of the chip at different power levels are obtained. The accuracy of the modeling method is verified by comparing the actual DPI test results of the board. Similarly, the comparison of the measured and simulated results of the frequency- and time-domain measurements at 50 MHz after inputting interference signals with different powers (Figure 19, Figure 20, Figure 21 and Figure 22) shows that the jitter of the chip’s output response increases as the input power is increased. When the input power is 10 dBM, the output signal is severely distorted, and the chip cannot work properly. It can be seen that the Sensi-Freq-Model can provide accurate output waveforms after disturbance, regardless of whether this disturbance is in the form of a small jitter or severe distortion.

**Figure 23:**
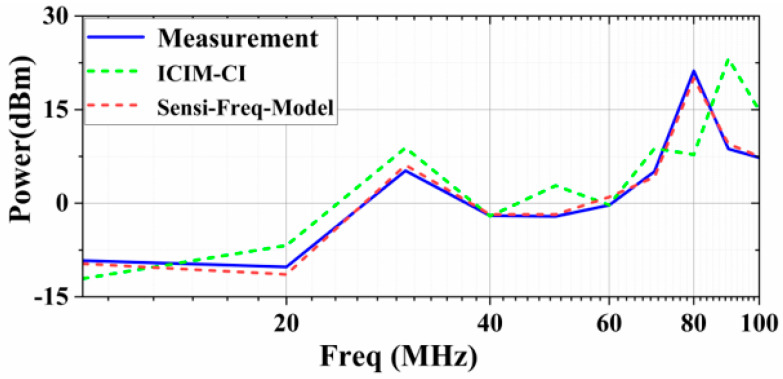
Comparative comparison of immunity prediction and DPI measurements for the two modeling approaches.

Figure 23 shows the comparison between the curves of the sensitivity level using the Sensi-Freq-Model method and the conventional ICIM-CI with the measured results when the condition of the immunity criterion ΔVout_p-p_ ≤ 30 mV is introduced. It can be seen that the op-amps increase their immunity to disturbances as the disturbance frequency increases, and this trend can be predicted using both modeling methods; it is obvious, however, that using the method proposed in this paper (Sensi-Freq-Model) is more accurate than the traditional ICIM-CI modeling method in terms of prediction accuracy.

#### 3.2.3. Discussion

Table 2 comprehensively demonstrates the comparison of the two modeling methods in the time and frequency domains in terms of modeling accuracy, modeling time, and whether cascade quantization simulation can be carried out. It can be seen that the Sensi-Freq-Model has obvious advantages in terms of modeling accuracy, modeling time, and support for quantization output and cascade simulation. Compared with ICIM-CI, using the method outlined in this paper (Sensi-Freq-Model) not only improves the modeling accuracy in the frequency domain by around 18.5 dB but also has significant advantages in terms of being able to output quantized waveforms at a single point and support cascade simulation, as well as improving the overall modeling time.

In contrast, the Sensi-Freq-Model method provides circuit designers with more flexibility in designing circuit boards by providing quantized waveform output from monitoring ports. Specifically, based on the specific waveforms output by the Sensi-Freq-Model, designers are able to not only work with different degrees of redundancy to meet diverse design needs but also adjust the immunity standard-setting guidelines for the chips on the board without having to rebuild the chip immunity model. In addition, designers can further optimize the board layout using the actual immunity waveform output data provided by the Sensi-Freq-Model. This form of layout adjustment based on real-world data is difficult to achieve in traditional behavioral-level black-box models of frequency-domain conduction immunity. With this approach, immunization problems in circuit design can be more accurately addressed and solved, improving the reliability and efficiency of the design.

## 4. Conclusions

In this study, we validate the proposed X-parameter-based IC frequency-domain conduction sensitivity modeling method, the Sensi-Freq-Model, by comparing simulation and real cases, and the results prove its effectiveness and accuracy in describing and predicting the conduction sensitivity of ICs. Compared with the traditional ICIM-CI modeling method, the Sensi-Freq-Model significantly reduces the time required for modeling and achieves a reduction of 18.5 dB in normalized mean square error (NMSE) in the frequency domain, which demonstrates its advantages in terms of efficiency and accuracy. In addition, the method provides quantifiable simulation results in the time domain, supporting the need for quantitative simulation of circuit board cascades and enhancing its application scope and utility. The Sensi-Freq-Model’s modeling process relies on only unclassified information and quickly obtains highly accurate conduction immunity predictions from measurements alone over a wide range of frequencies, even in the absence of a full-impedance model of the integrated circuit and the surrounding PCB. Even in situations where a full impedance model of the IC and its surrounding PCB is not present, interference information can be accurately captured in the time-frequency domain over a wide range of frequencies, without being limited by the criteria for determining chip susceptibility. In light of the above, the Sensi-Freq-Model not only meets the needs of most IC terminal users to predict potential EMI in electronic devices but also provides an efficient and accurate modeling tool for circuit design and EMC analysis.

## Figures and Tables

**Table 1:** Output type and description of the operational amplifier after interference.

Case	Criteria	IC Performance Level	Description
Figure 4	ΔVout_p-p_ ≤ 13.2 mV	Class A_IC_	Normal output
Figure 5		Class A_IC_	All monitored functions of the IC perform within the defined tolerances during and after exposure to disturbance.
Figure 6		Class C_IC_	The output waveform experiences distortion or jitter. The IC does not perform within the defined tolerances during exposure and does not return to normal operation. It returns to normal operation via manual intervention.
Figure 7		Class C_IC_	The output waveform experiences serious distortion or jitter. The IC does not perform within the defined tolerances during exposure and does not return to normal operation by itself. It returns to normal operation via manual intervention.

**Table 2:** Comparison of the NMSE and modeling times of different models.

Signal Type	Modeling Method	NMSE (dB)	Modeling Time	Supports Cascade Quantization Simulation
Time domain	Sensi-Freq-Model	−30.92	21 s	Yes
	ICIM-CI	No waveform output		No
Frequency domain	Sensi-Freq-Model	−31.3352	0.58 h	Yes
	ICIM-CI	−12.7982	8.3 h	No

## Data Availability

The original contributions presented in the study are included in the article.
